# Proteomic analysis during larval development and metamorphosis of the spionid polychaete *Pseudopolydora vexillosa*

**DOI:** 10.1186/1477-5956-7-44

**Published:** 2009-12-14

**Authors:** Flora SY Mok, Vengatesen Thiyagarajan, Pei-Yuan Qian

**Affiliations:** 1 KAUST Global Partnership Program, Department of Biology/Atmospheric, Marine and Coastal Environment Program, The Hong Kong University of Science and Technology, Hong Kong SAR, China; 2 Swire Institute of Marine Science/School of Biological Sciences, The University of Hong Kong, Hong Kong SAR, China; 3 CAS/SAFEA International Partnership Program for Creative Research Teams, South China Sea Institute of Oceanology, Chinese Academy of Sciences, China

## Abstract

**Background:**

While the larval-juvenile transition (metamorphosis) in the spionid polychaete *Pseudopolydora vexillosa *involves gradual morphological changes and does not require substantial development of juvenile organs, the opposite occurs in the barnacle *Balanus amphitrite*. We hypothesized that the proteome changes during metamorphosis in the spionids are less drastic than that in the barnacles. To test this, proteomes of pre-competent larvae, competent larvae (ready to metamorphose), and juveniles of *P. vexillosa *were compared using 2-dimensional gel electrophoresis (2-DE), and they were then compared to those of the barnacle.

**Results:**

Unlike the significant changes found during barnacle metamorphosis, proteomes of competent *P. vexillosa *larvae were more similar to those of their juveniles. Pre-competent larvae had significantly fewer protein spots (384 spots), while both competent larvae and juveniles expressed about 660 protein spots each. Proteins up-regulated during competence identified by MALDI-TOF/TOF analysis included a molecular chaperon (calreticulin), a signal transduction regulator (tyrosin activation protein), and a tissue-remodeling enzyme (metallopeptidase).

**Conclusions:**

This was the first time to study the protein expression patterns during the metamorphosis of a marine polychaete and to compare the proteomes of marine invertebrates that have different levels of morphological changes during metamorphosis. The findings provide promising initial steps towards the development of a proteome database for marine invertebrate metamorphosis, thus deciphering the possible mechanisms underlying larval metamorphosis in non-model marine organisms.

## Background

Marine invertebrates have a biphasic life cycle that includes a pelagic larval stage that is morphologically distinct from the adult form [1]. Their larvae typically metamorphose into juveniles or adults simultaneously with or directly following settlement out of the water column; they will subsequently colonize and structure the benthic communities [2]. Marine industries also target settlement and metamorphosis processes when developing methods to eliminate species that deteriorate infrastructure and commercial resources by biofouling man-made surfaces or interfering with aquaculture [3,4].

In general, common features of marine invertebrate metamorphosis include tissue remodeling and differentiation that are under hormonal or transcriptional control and are mediated by differential gene and protein expressions [5]. However, the specific mechanisms of metamorphosis vary from species to species [6,7]. For example, since the metamorphic transitions in different taxa might have evolved under different selective pressures, their temporal orders of metamorphic phases are different [7]. In cnidarians, bryozoans, and sponges, settlement precedes differentiation of adult structures; while in colonial ascidians and molluscs, adult morphogenesis takes place before settlement [8]. In sea urchins, morphological changes during metamorphosis are more drastic and many of the adult structures are generated from undifferentiated cells set aside in the larva [9]. In gastropods, metamorphosis is a series of dynamic events including both reorganization of larval tissues and differentiation of their primordial [10]. The morphological, behavioural, and differential gene expressions during larval metamorphosis have been well-studied among some marine invertebrates [11-13], but relatively fewer studies focus on comparing the commonalities and differences underlying the metamorphic processes between different marine invertebrates [7]. Even fewer studies focus on the protein expressions in marine invertebrate metamorphosis [14].

The rapid development of proteomics technology allows characterization of biological processes and mechanisms by quantitative protein-level measurements of gene expressions [15], even when there is little information on protein identity, which often occurs in non-model organisms [16]. It allows us to compare the differential expression of proteins during the process of metamorphosis among different marine invertebrates. This can provide insights for deciphering the intrinsic factors that determine their timing of achieving competence, the molecular changes that initiate metamorphosis, and the mechanisms that lead to completion of metamorphosis [17-19].

A previous study on proteome changes during marine invertebrate metamorphosis found a dramatic reduction in the number of proteins expressed after settlement but did not see extensive *de novo *synthesis of proteins during larval metamorphosis in the barnacle *Balanus amphitrite *[20]. Unlike the metamorphosis in barnacles, where adult structures are formed only after settlement, adult structures begin to develop prior to settlement in most families of polychaetes, including the Spionidae. Spionid polychaetes are abundant and important members of the estuarine or shallow marine benthic communities [21-24]. They are common dietary items to fish and shorebirds [22,23], and at such high densities that they exclude other benthic species including commercially important bivalves [24]. Most spionid polychaetes have a biphasic life cycle, in which their larvae settle on hard substrates or soft sediments and spontaneously metamorphose into benthic juveniles [25]. Adult traits are commonly expressed in larval stages of spionids and are carried over through the metamorphosis [21,26]. In barnacles, substantial re-organization of internal tissues occurs during settlement and metamorphosis [20], while in spionids, morphological metamorphosis generally involves little more than the loss of swimming chaete, body elongation (addition of new segments at the posterior end), and re-organization of palps [26]. We therefore hypothesized that protein expression before and after metamorphosis in the spionid polychaete *Pseudopolydora vexillosa *would be relatively similar. We also expected little de novo synthesis of proteins during metamorphosis and less drastic reduction in protein number in metamorphosed *P. vexillsoa *that those in metamorphosed *B. amphitrite*.

To test our hypotheses, we carried out a comparative proteomic analysis of pre-competent larvae, competent larvae, and newly metamorphosed juveniles of *Pseudopolydora vexillosa*. MALDI-TOF/TOF MS was then used to identify the proteins that are differentially and abundantly expressed during larval competence. We compared the general patterns revealed in this study with ones described by Thiyagarajan & Qian (2008) [20].

## Results

### The proteome of *Pseudopolydora vexillosa* larvae

**Figure 1:**
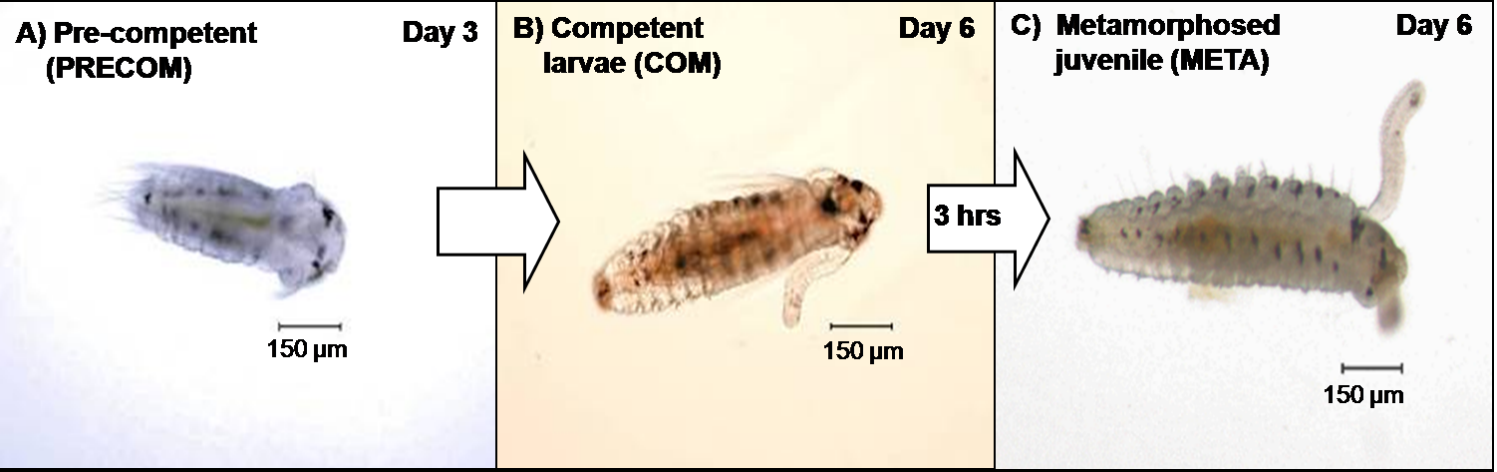
**Developmental stages of the spionid *Pseudopolydora vexillosa***. Three developmental stages were chosen for proteomic analysis; (A) pre-competent larvae, (B) competent larvae, and (C) newly-metamorphosed juveniles.

**Figure 2:**
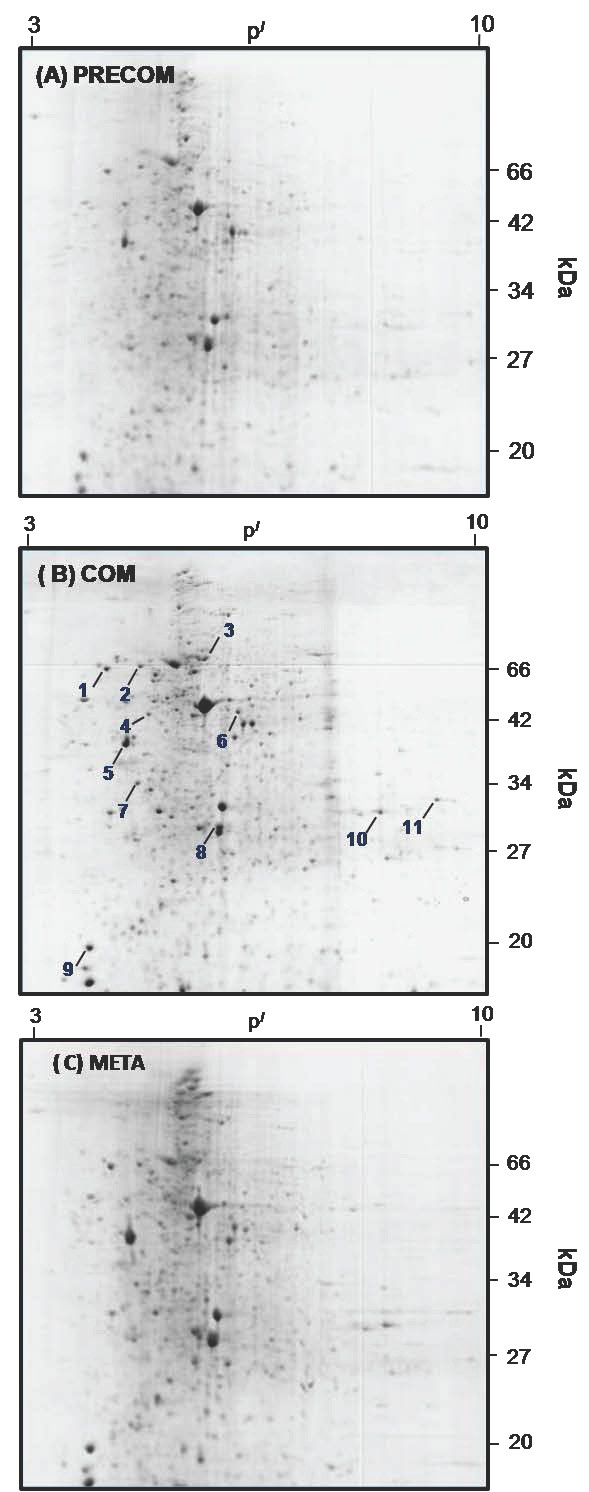
**Representative 2-DE gel images showing the proteomes of (A) pre-competent larvae, (B) competent larvae, and (C) newly-metamorphosed juveniles of *Pseudopolydora vexillosa***. The 1^st ^dimension was performed on pH 3-10 linear IPG strips; the second dimension was run on 12.5% acrylamide gels. Gels were stained with Colloidal Coomassie Blue stain. The protein load per gel was about 400 μg. Marked protein spots were identified by MS and their details are listed in Table 1.

**Figure 3:**
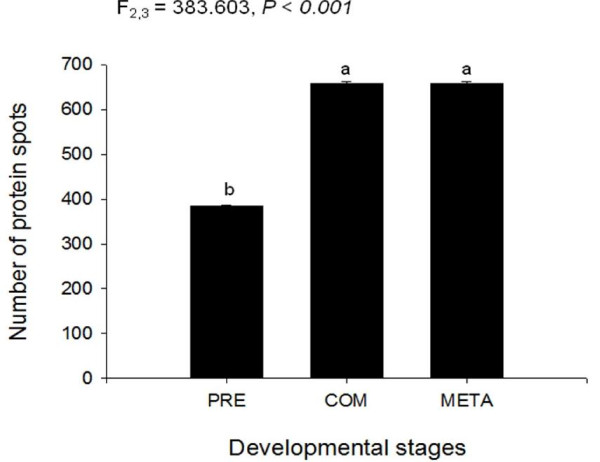
**The mean numbers of protein spots expressed in *P. vexillosa *pre-competent larvae, competent larvae and newly-metamorphosed juveniles. **Each bar represents the mean (± S.D.) of 2 replicate 2-DE gels. Letters indicate significant differences by Tukey HSD posthoc test.

Most of the protein spots in the 3 developmental stages (pre-competent larvae, competent larvae, and newly metamorphosed juveniles, Fig. 1) were in the range of p^*I *^4-7 and concentrated in the molecular weight range of 14-90 kDa (Fig. 2). The number of protein spots varied significantly between stages (F_2,3 _= 383.603, *p *< 0.001); pre-competent larvae had significantly fewer protein spots (Tukey HSD: **p **< 0.05; 384 ± 1; mean ± S.D.) than competent larvae (657 ± 4; mean ± S.D.) and newly metamorphosed juveniles (658 ± 5; mean ± S.D.) (Fig. 3).

**Table 1:** Proteins of *Pseudopolydora vexillosa *larvae showing differential expressions when attaining competence.

No.	Acc. No.	Proteins identified	Mascot score (No. peptides matched)	kDa	**p**^*I*^	Fold Change	Unique peptides
1	gi|47550939	Calreticulin	78(6)	49	4.2	1.9	GLQTSEDAR VHVIFNYK KVHVIFNYK VHVIFNYKGK EEFNDGDGWKSR IMFPPDICGPGTK
2	gi|32967463	90-kDa heat-shock protein precursor	105(4)	66	5.1	1.56	DYVTRMK VIKDILDK TKPIWMR ELISNSSDALDKIR
3	gi|4468655	Cytoplasmic intermediate filament protein	86(10)	69	5.7	3.86	ELAALAYR LLEGEENR FLEAQNRK DMQDLNER LQDSLNKAR LLDDAEKEK KLLEGEENR VRFLEAQNR KDMQDLNER AMYQAELDEAR
4	gi|46909257	ATP synthase beta subunit	303(8)	37	5.1	1.62	IQWFLSQ IGLFGGAGVGK VVDLLAPYAK GGKIGLFGGAGVGK AHGGYSVFAGVGER VSLVYGQMNEPPGAR GIAELGIYPAVDPLDSNSR IPSAVGYQPTLATDMGTMQER
5	gi|47117349	Tropomyosin	149(8)	33	4.6	1.59	ATEAERQVVK LAITEVDLER KLAITEVDLER LEEASKAADESER EVDRLEDELLAEK QITQLESDVGALQRR ENAQDLAEQMEQKLK LTTTENNFDVANEQLQEANVK
6	gi|59894747	Actin	66(8)	42	5.4	1.60	CDVDIRK IIAPPERK AGFAGDDAPR GYSFTTTAER AVFPSIVGRPR DSYVGDEAQSKR QEYDESGPSIVHR SYELPDGQVITIGNER
7	gi|61651838	Tyrosine 3-monooxygenase activation protein	120(8)	29	4.8	2.89	NLLSVAYK IISSIEQK DEDLSVEER TAPQDAEEEK YDEMVESMK DSTLIMQLLR AASDIAMTELPPTHPIR LAEQAERYDEMVESMK
8	gi|178045	Gamma-actin	442(13)	26	5.7	1.53	CDVDIRK IIAPPERK DLTDYLMK IKIIAPPER GYSFTTTAER KITALAPSTMK QEYDESGPSIVHR DLTDYLMKILTER GYSFTTTAEREIVR LDLAGRDLTDYLMK QEYDESGPSIVHRK SYELPDGQVITIGNER DLYANTVLSGGTTMYPGIADR
9	gi|11596085	Troponin C	50(2)	17	4.3	1.5	ELKEIFR MSLNCKADWLEK
10	gi|6981214	Matrix metallo-peptidase 7	54(8)	30	7.7	-3.61	FFGLPETGK KFYLHDSK FYLHDSKTK VMEIMQKPR MAAMRLTLFR MWSMQIPLNFK MWSMQIPLNFKR ALRMWSMQIPLNFK
11	gi|467525	T cell receptor delta pre-chain	53(8)	31	9.5	5.53	EPKVYAEK NGTNVACLVK EIGSAIVISPSK YSINFQKEAK VNMMSLTVLGLR YLCALSEPNIR VNMMSLTVLGLRK SGAINFLLTAKLFFS

The proteins listed above expressed abundantly across 3 developmental stages but had at least a 1.5-fold change in spot volume at the competent larval stage. Mascot scores > 50 are considered significant.

**Figure 4:**
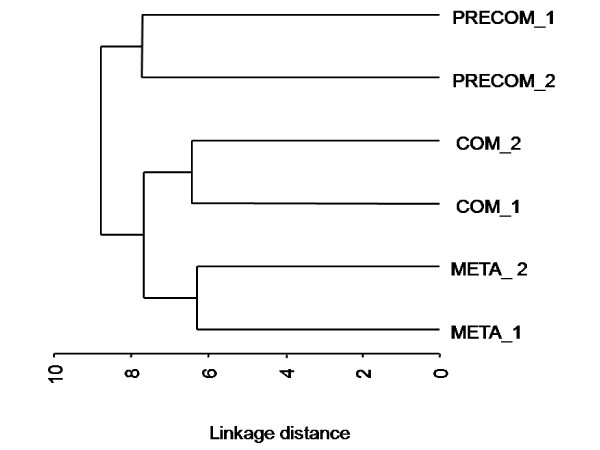
**A dendogram for all protein spots in 3 developmental stages. **It is constructed using Euclidian distances summed over spots and the Ward's method for all protein spots in 6 samples. PRECOM: pre-competent larvae, COM: competent larvae, META: newly metamorphosed juveniles.

The dendrogram constructed from the Euclidian distances showed that replicates within each developmental stage grouped together, indicating high reproducibility between them (Fig. 4). The proteome of pre-competent larvae distinctly separated from the other two stages, which were grouped together (Fig. 4).

### Differential protein expression patterns among developmental stages

**Figure 5:**
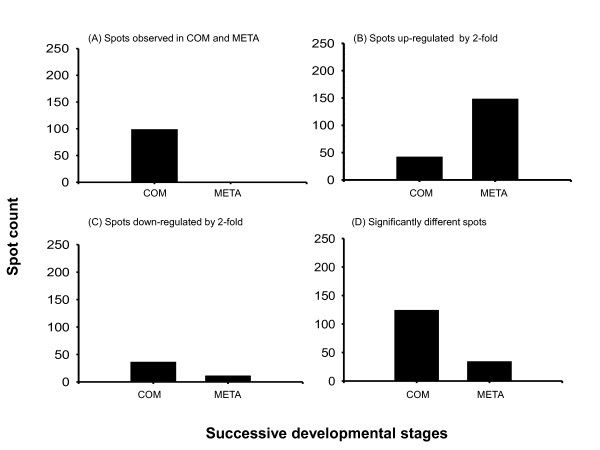
**Differential expression of proteins during the development of spionid *Pseudopolydora vexillosa *larvae. **COM: competent larvae, META: newly metamorphosed juveniles. (A) Number of protein spots observed in each stage. (B) Number of protein spots showing a 2-fold increase in spot volume between successive stages, and (C) number of protein spots that showed a 2-fold decrease in spot volume between successive stages. (D) Number of spots that showed a significant difference in both their presence/absence and their spot volumes between successive stages by Student's *t *test (*p *< 0.02), n = 2.

Ninety-nine new protein spots, concentrated in the p^*I *^range of 4.5 to 7, appeared in the competent larvae that were not evident in pre-competent larvae; no new protein spots were detected in metamorphosed juveniles (Fig. 2, 5A). A visual comparison of the protein profiles from competent larvae and metamorphosed juveniles showed that the positions of the protein spots in these two stages were unchanged (see Fig. 2B, 2C). Between the competent larvae and the newly-metamorphosed juveniles, 149 proteins were up-regulated and 11 were down-regulated (Fig. 5B, 5C). The protein gels of the competent larvae showed 124 protein spots that were significantly different (in terms of both presence and volumes of the protein spots) from those of the pre-competent larvae, but only 34 spots that were significantly different from the newly-metamorphosed juveniles (t_2 _= 6.967, *p *< 0.02, Fig. 5D).

### Identification of selected proteins

Sixty-eight spots showing differential changes in spot volumes of at least 1.5 folds were excised from a total of about 660 reproducibly detected spots from the 2-DE ge1 of competent larvae. Protein identification using MS from the proteomes of *Pseudopolydora vexillosa *is limited by the lack of sequenced genome information; therefore, we could identify only 11 out of the 68 protein spots (Fig. 2B, Table 1). These differentially-expressed spots included proteins involved in signal transduction, stress response, structural remodeling, and metabolism, including a molecular chaperone (calreticulin, spot#1), a signal transduction regulator (tyrosin 3-monooxygenase activation protein, spot#7), and a tissue-remodeling enzyme (matrix metallopeptidase 7, spot#10) (Table 1).

## Discussion

The application of proteomics to study developmental changes in marine invertebrates has only recently been emerging [27,28]. In this study, we provide information for the first time on the proteomic changes during the larval development and metamorphosis of the spionid polychaete Pseudopolydora vexillosa. Similar data is available for the crustacean *Balanus amphitrite *(the only other such data currently available) [20], giving us the opportunity to compare the proteomic changes occurring at metamorphosis between these invertebrates.

There was a remarkable increase in the number of new protein spots in the competent larvae compared to the pre-competent larvae, in both *Pseudopolydora vexillosa *and *Balanus amphitrite*. These new proteins were probably necessary for the development of structures and tissues for competent marine invertebrate larvae to be capable of achieving metamorphosis and surviving a benthic existence after metamorphosis [29]. In most spionid larval development, adult features including neurochaete, sensory structures (e.g. microvilli on the prostomium), and feeding structures (e.g. palps with ciliated grooves) are accumulated and become differentiated in the competent stage; these structures are then retained or become functional during metamorphosis and in the juvenile stage [25,30,31]. Barnacle larvae also develop new structures and carry out new functions when attaining competency, such as, transforming their naupliar swimming limbs into specialized walking limbs, shifting their heads into new configurations, and bringing their thoracic limbs into function [32]. These new developments explain the trend of protein accumulation at the transition from pre-competency to competency that is maintained through metamorphosis in both *P. vexillosa *(Fig. 3, 5A) and in *B. amphitrite*.

Protein synthesized during the period that larvae are attaining competence might be used for environmental-cue reception and habitat selection prior settlement. For instance, adenylate cyclase modulates larval settlement ability during the swimming cyprid stage in barnacles [33]. A putative chemosensory Gαq gene encoding a G protein has been recently localized on the palp sensory cells of another spionid *Dipolydora quadrilobata *[34]. However, in this study, we were unable to identify any sensory or receptor proteins using MS. These proteins, together with transcription factors and hormones, are important regulatory proteins but with very low abundance. It is possible that these proteins are masked by proteins with higher abundance on the gel due to protein co-migration and differential protein migration [35]. Therefore, their expression levels were neither detected by protein spot pattern analysis nor effectively detected in MS. To reveal these proteins, in the future we need to employ technologies like proteome pre-fractionation and narrow-range isoelectrofocusing, to reduce sample complexity and increase detection of low abundance proteins in the samples [36,37].

The proteomes of the competent larvae and the metamorphosed juveniles of *Pseudopolydora vexillosa *were highly similar in terms of the number and the major protein spots expressed, with no new protein spots being observed after metamorphosis (Fig. 2B, 2C). It is possible that new protein spots were masked by more abundant protein spots on the 2D gel due to protein co-migration [35], and hence no new protein spots were observed. To better separate the protein spots and investigate the expressions of low abundant proteins, 2DE with narrow-range IPG strips and LC MS/MS will be carried out in our future studies. This might also suggest that during metamorphosis in these species, no new proteins have been synthesized. Metamorphosis in many marine invertebrates typically involves the loss of larval characters, followed by the emergence of juvenile characters [30]. However, in the case of spionid polychaetes, very little structural generation happens after larvae shed their larval setae and most ciliary bands during metamorphosis. Adult structures, such as neurochaete, sensory structures (e.g. microvilli on the prostomium), and feeding structures (e.g. palps), have already been developed and differentiated when spionid larvae achieve competence [22,25,38]. A previous hypothesis raised by Hadfield et al. [29] explained that marine invertebrate larvae were naturally selected to have speedy metamorphosis to minimize the time when they were most vulnerable to predation, that is, the period after losing their larval structures and before they can completely settle in the new habitat. In another developmental study on the ubiquitous fouling polychaete *Hydroides elegans*, *de novo *protein transcription and translation are not required during the metamorphic process until juvenile growth begins [39], which suggested that protein synthesis might not be necessary during larval metamorphosis in polychaetes.

Different trends were observed in the change of total number of protein spots at metamorphosis in *Pseudopolydora vexillosa *and *Balanus amphitrite*. In *P. vexillosa*, the total number of protein spots remained similar during the transition between competent and metamorphosed stages (Fig. 3); on the other hand, the total number of spots decreased by 50% after competent cyprids metamorphosed into young juveniles in *B. amphitrite *[19]. It is likely that some proteins in *B. amphitrite *are no longer required to be maintained at a high level, or may even need to be degraded, upon metamorphosis. For instance, barnacle larvae invest heavily in their brains and sensory organs during their extensive search for habitat/cues prior to settlement; their brains are small in size at naupliar stages, become well developed at competence, and then reduce in size and almost disappear in adult barnacles [40]. Degeneration of cephalic sensory structures after attachment could partially account for the reduction in the number of proteins during attachment and metamorphosis of *B. amphitrite*. Spionid polychaetes experience less structural loss during metamorphosis than the barnacles, because spionid juvenile structures that were built before competence would be retained during metamorphosis [22,25]. We speculated that post-translational modifications instead of new protein synthesis controlled the morphological modifications involved in metamorphosis of *P. vexillosa *[14], which may also explain why there was little change in the total proteome before and after metamorphosis in this species.

Among the proteins identified by mass spectrometery, calreticulin, tyrosin 3-monooxygenase activation protein, and matrix metallopeptidase were up-regulated during the transition from the pre-competent to the competent larvae of *Pseudopolydora vexillosa*. Calreticulin is a molecular chaperone for glycoproteins in the endoplasmic reticulum of eukaryotic cells. It is involved in calcium homeostasis and immune functions and plays a significant role in the folding of nascent polypeptides [41]. To date, calreticulin has been shown to be involved in the anti-virus immune system of the Chinese shrimp and is up-regulated after the shrimp is challenged by white-spot-syndrome virus [42,43]; it has also been detected in the proteome of marine mussels *Mytilus spp *[44]. This study is the first to report the presence and up-regulation of calreticulin in competent polychaete larvae. Since increased levels of calreticulin can enhance stress tolerance by preventing the accumulation of stress proteins and targeting damaged proteins for proteolytic degradation [44,45], its up-regulation may help the metamorphosing larvae to go through the highly stressful transition from pelagic to benthic existence. Tyrosin 3-monooxygenase activation protein is a key regulator in signal transduction, cytoskeletal organization, and cellular metabolism [46]. This protein binds to apoptotic factors in the marine sponge *Geodia cydonium *to prevent polychlorinated biphenyls-induced apoptosis [47], and its levels significantly increase upon challenge by viral infection in shrimps [42,48]. This study is also the first to discover up-regulation of this protein during larval metamorphosis in marine polychaetes; the up-regulation observed in this study might be due to oxidative metabolism during development, since ATP synthase beta subunit was also up-regulated. Matrix metallopeptidase is required in the extracellular matrix to process tissue remodeling, cell growth and differentiation, and embryonic development [49]. This enzyme is involved in the onset of intestine regeneration in sea cucumber [50] and hydrolyzed gelatin during all 6 naupliar stages of the barnacle *Balanus amphitrite *[49]. So far, Myohara et al. [51] have provided the only report about an up-regulation of its gene in the regenerating fragments of an oligochaete. Further evidence is required to characterize the involvement of matrix metallopeptidase 7 in tissue remodeling in the competent larvae of *P. vexillosa*. To further clarify the functions of these three proteins and the implications of their up-regulations during the transition between the pre-competent and the competent stage in *P. vexillosa*, the next steps will be to sequence their peptides, design degenerate primers from the sequences, and amplify the specific genes, followed by subsequent sequencing and function validation of the corresponding genes.

## Conclusions

This was the first study to use 2-D gel electrophoresis to investigate the global protein expression differences during settlement and metamorphosis in a polychaete, one of the very important taxa in marine benthic ecosystem. The total proteomes of *Pseudopolydora vexillosa *at the competent larval stage and at the newly-metamorphosed stage were highly similar, and the total number of protein expressed in *P. vexillosa *larvae doubled as the larvae developed from pre-competent to the competent stage. Eleven proteins up-regulated at the competent stage were identified, which include calreticulin, tyrosin 3-monooxygenase activation protein, and matrix metallopeptidase. This was also the first attempt to compare the changes in protein expression patterns occurred during larval-juvenile transition between polychaete (Phylum: Annelida) and barnacle (Phylum: Arthropoda). The proteome data generated in this study provides a foundation for future comparative proteomic analyses and for the development of a proteome database for marine invertebrate larvae. Finally, by identifying some of the proteins up-regulated during competency, we have identified some promising targets for future analyses of protein function in the metamorphosis of spionid polychaetes.

## Methods

### Study organism and larval sample collection

Adult subtropical spionid *Pseudopolydora vexillosa *were collected from subtidal soft-bottom substrates in Sai Kung, Hong Kong (22°25'N, 114°17'E), and maintained in laboratory cultures. From these cultures, pre-competent larvae, competent larvae, and newly metamorphosed juveniles of *Pseudopolydora vexillosa*, (Fig. 1) were collected according to Mok et al. [52] Briefly, larvae are released from the adult's tube when they have 3 fully developed setigers then develop for 6 days at 28°C until they become competent at the 13-14 setiger stage. Larvae will then settle and metamorphose within 24 hours when provided sediment substratum [22].

Over a 24-hour period, we collected the newly-released larvae at the 3-setiger stage using an 80-μm mesh. Larvae were reared in multiple batches (2 larvae ml^-1^) in FSW under constant temperature (27°C). Water was changed and larvae were fed the diatom *Chaetoceros gracilis *Schutt at a concentration of ~ 10^5 ^cells ml^-1 ^every alternate day. Pre-competent larvae were collected for processing at day 3 (7-9 setigers) and competent larvae were collected at day 6 (12-14 setigers). Autoclaved sediment (particle size < 60 μm) was provided as a substratum for competent larvae to settle and metamorphose in; newly metamorphosed juveniles were removed from the sediment after 2 h by gently probing at their tubes and were collected for sample preparation immediately.

Five separately-collected batches of pre-competent larvae, competent larvae, or juveniles were each pooled into sample tubes so that enough protein could be extracted for one 2-D gel electrophoresis. Replicates of pooled samples from each developmental stage of *Pseudopolydora vexillosa *were prepared for proteomic analysis; sample preparation followed methods of Thiyagarajan & Qian [20], with slight modifications. Each batch of larvae/juvenile, containing 300-600 larvae or 200-300 juveniles, was rinsed quickly with 15ppt filtered sea water (FSW) and placed in lysis buffer consisting of 7M urea, 2M thiourea, 4% 3-[(3-Cholamidopropyl)dimethylammonio]-2-hydroxy-1-propanesulfonate (CHAPS), 40mM 1,4 dithiothreitol (DTT), and 2% Bio-Lyte 3/10 ampholyte, and then fast-frozen in gas-phase liquid nitrogen and stored in -80°C until protein extraction. For protein extraction, samples were first sonicated (Branson Digital Sonicator 250) on ice to solubilize proteins, using ten 5s blasts of 15% amplitude, with 10s pauses between blasts; this step was designed to prevent over-heating the proteins due to sonication [53]. Then samples were centrifuged at 13,000rpm for 20 min, the pellet was discarded, and the protein supernatant was stored at -80°C until further analysis. The protein content was determined following a modified Bradford method previously described [54].

### 2-Dimensional gel electrophoresis (2-DE)

All preparation kits and instruments used in the 2-DE analysis were purchased from Bio-Rad (Hong Kong, China) and all chemicals from Bio-Rad, Merck, Fluka, or Sigma (Hong Kong, China), unless otherwise specified. The sample preparation and electrophoresis process followed the methods in Thiyagarajan & Qian [20] with modifications. All samples were desalted using a 2-D cleanup kit and solubilized in rehydration buffer (7M urea, 2M thiourea), 2% CHAPS, 40mM DTT, 0.2% Bio-Lyte 3/10 ampholyte and 1% Bromophenol blue). 300 μl, containing 400 μg protein determined by modified Bradford method as described by Ramagli and Rodriguez [54], was applied to 17 cm immobilized pH gradient (IPG) strips, pH 3-10 (linear), for ~ 14h active rehydration and then subjected to isoelectrical focusing (IEF) using a Protean IEF Cell. Focusing was performed according to the following regime: 250 V for 20 min, followed by a gradient from 1000 to 10000V over 2.5 hrs, adding up to a total of 40000Vh. The current did not exceed 50 μA per strip. After IEF, the IPG strips were equilibrated for 15 min in equilibration buffer I [6M urea, 2% SDS, 0.05M Tris-HCL (pH 8.8), 50% glycerol and 2% (w/v) DTT] followed by 15 min in buffer II (same as buffer I but containing 2.5% iodacetamide instead of DTT). To capture the second dimension, the equilibrated IPG strips were placed on top of 12.5% sodium dodecyl sulphate-polyacrylamide gels (18x18cm) and sealed with 0.5% w/v agarose and bromophenol blue. The running buffer system was the standard Laemmli buffer for SDS-PAGE. Aliquots (20 μl) of protein standards including trypsin inhibitor (20 kDa), triosephosphate isomerase (27kDa), thioredoxin reductase (34kDa), maltose-binding protein 2 (42kDa) and serum albumin (66kDa) were added. The gels were run at 20°C at a maximum of 24mA per gel for approximately 5 h until bromophenol blue reached the bottom of the gel. Gels were stained with freshly-prepared G-250 Coomassie Brilliant Blue for 36 h [55].

### Analysis of spot patterns in gels

The gels were scanned using a GS-800 Scanner (Bio-Rad) at an optical resolution of 400dpi. The scanned gels were analyzed with the PDQuest software v.8 (Bio-Rad) according to the manufacturer's protocols. By aligning the images using Land Marking, corresponding proteins were matched to each other. Gel comparisons between different developmental stages were performed by modeling the protein spots into a 3-dimensional Gaussian distribution and determining the maximum absorption after raw image correction and background subtraction. Spot intensities were normalized so that the total density in each gel image was equal, and analysis was performed using quantitative and qualitative modes. Automatic spot detection in each gel was verified by visual inspection to ensure that the image patterns were as similar as possible to the original gel. A dendogram was constructed from the squared Euclidian distances, the squared differences in spot volumes summed over the number of spots, between the replicates within each developmental stage using hierarchical clustering and Ward's method. Changes in the normalized protein spot volumes in proteomes were compared between successive developmental stages to determine the differential expression of proteins during larval development, competence, and metamorphosis. The numbers of protein spots observed in the competent larvae and in the metamorphosed juveniles were calculated using the qualitative analysis option in the PDQuest software. Only protein spots showing at least a 2-fold change, calculated by the quantitative analysis option in the PDQuest software were defined as up- or down-regulated, in order to compensate for the technical variability between replicates. Both the presence or absence and the up- or down-regulation of spot volume between each two successive stages were compared with a Student's *t *test at α = 0.05 level using the PDQuest software. The total number of protein spots expressed in each developmental stage was compared with a single-factor analysis of variance (ANOVA) at α = 0.05 level in SPSS software.

### Trypsin digestion and Mass Spectrometry

Sixty-eight spots that showed a differential increase in spot volume by at least 1.5 times in the competent larval stage compared to the pre-competent stage, and fulfilled the following criteria: 1) they were reproducibly detected among the replicates, 2) they had adequate volume for successful generation of MS/MS spectra, and 3) they represented the entire biochemical range of the proteome in terms of both molecular weight (Mr) and isoelectric points (p^*I*^), were selected for tandem mass spectrometry (MS/MS). These spots were excised from 1 replicate gel and digested according to the method described by Thiyagarajan & Qian [21]. Briefly, gel plugs were washed twice with ddH_2_O for 15 min and twice with H_2_O/CH_3_CH (1/1 v/v) and then placed in 100% CH_3_CN. The gel plugs were dried using a SpeedVac centrifuge before 10 μl of trypsin solution (Promega) containing 20 ng/μl sequencing-grade trypsin in 20mM NH_4_HCO_3 _buffer was added. After rehydration with the enzyme solution, the buffer solution was added to cover the gel pieces, and digestion was allowed to proceed for ~ 16h at 37°C. Peptides were extracted using H_2_O/CH_3_CN/trifluoroacetic acid mixture (80:20:1). These fractions were pooled, dried in a vacuum centrifuge, and then re-dissolved in 50 μl of 5% v/v formic acid. The digests were cleaned up with ZipTip (Millipore, USA) and subjected to analysis by the MALDI-TOF/TOF mass spectrometer (4700 Proteomics Analyzer, Applied Biosystems).

### Analysis of peptide sequences

The Peptide Map Fingerprints (PMF) and MS/MS information obtained from the MS process were automatically submitted through MASCOT to the NCBI non-redundant database (Metazoa/Other Metazoa). Mass searches were performed using mass tolerance settings of ± 100 pm for the PMF and ± 0.2 Da for the MS/MS spectra. To determine the MS-fit, mass searches were carried out via a mass range between 1 to 100kDa. For PMF, it was assumed that peptides were monoisotopic, oxidized at the methionine residues and carbamidomethylated at cysteine residues. Up to one missed trypsin cleavage was allowed, although most matches did not contain any missed cleavages. The score threshold for peptide was suggested by MASCOT at 50 (*p *< 0.05).

## Competing interests

The authors declared that they have no competing interests.

## Authors' contributions

PYQ conceived the study, designed, and coordinated the experiments. FSM involved in the experimental work, including sample collection, sample preparation, electrophoresis, and data analysis. VT established the procedures of 2DE, provide helpful suggestions to the data analysis and the manuscript. FSM drafted and finished the final version of manuscript. PYQ provide critical revisions leading to the final version of manuscript. All authors read and approved the final manuscript.
